# The Social Effects of an Intergenerational E-Mail Pal Program

**DOI:** 10.1093/geroni/igab046.686

**Published:** 2021-12-17

**Authors:** Kristi Lise, Jillian Minahan Zucchetto, Karen Siedlecki

**Affiliations:** 1 CUNY Queens College, New Rochelle, New York, United States; 2 Fordham University, United States; 3 Fordham University, Bronx, New York, United States

## Abstract

Ageist attitudes and loneliness negatively impact both younger and older adults (e.g., Sun et al., 2019). This study utilized a randomized waitlist-control design to investigate the effects of a six-week intergenerational e-mail pen pal program on loneliness in younger and older adults and ageism in younger adults. Thirty-three younger adults (18-30 years) and 28 older adults (over age 65) completed an online survey assessing ageist attitudes, loneliness, well-being, and other individual differences. One week after completing a baseline survey, 17 email pen pal pairs began the six-week e-mail intervention. Participants repeated the survey one week after the completion of the intervention (which was eight weeks after the baseline for the control participants). Analyses showed that at baseline, younger adults (M=2.41, SD=.76) reported higher levels of loneliness compared to older adults (M=1.65, SD=.77), t(59) = 3.85, p < .001. Repeated measures ANOVAs showed that the intervention did not have a significant effect on ageism or loneliness in either younger or older adults. However, the effect size of the intervention for loneliness among older adults was moderate to large (η2= .07). Descriptive statistics indicated that older adults in both the intervention and control groups experienced an increase of loneliness during the post-test. However, the older adults in the intervention group experienced less of an increase compared to older adults in the control group. This suggests that the intervention may have buffered the increase in loneliness that older adults may experience during the winter months and during the onset of the COVID-19 pandemic.

